# Two VHH Antibodies Neutralize Botulinum Neurotoxin E1 by Blocking Its Membrane Translocation in Host Cells

**DOI:** 10.3390/toxins12100616

**Published:** 2020-09-27

**Authors:** Kwok-Ho Lam, Kay Perry, Charles B. Shoemaker, Rongsheng Jin

**Affiliations:** 1 Department of Physiology & Biophysics, University of California, Irvine, CA 92617, USA; kwokhl@uci.edu; 2 NE-CAT and Department of Chemistry and Chemical Biology, Cornell University, Argonne National Laboratory, Argonne, IL 60439, USA; kperry@anl.gov; 3 Department of Infectious Disease and Global Health, Cummings School of Veterinary Medicine, Tufts University, North Grafton, MA 01536, USA; charles.shoemaker@tufts.edu

**Keywords:** botulinum neurotoxin, botulism, single-domain antibody, VHH, neutralizing epitope, antitoxin, membrane translocation

## Abstract

Botulinum neurotoxin serotype E (BoNT/E) is one of the major causes of human botulism, which is a life-threatening disease caused by flaccid paralysis of muscles. After receptor-mediated toxin internalization into motor neurons, the translocation domain (H_N_) of BoNT/E transforms into a protein channel upon vesicle acidification in endosomes and delivers its protease domain (LC) across membrane to enter the neuronal cytosol. It is believed that the rapid onset of BoNT/E intoxication compared to other BoNT serotypes is related to its swift internalization and translocation. We recently identified two neutralizing single-domain camelid antibodies (VHHs) against BoNT/E1 termed JLE-E5 and JLE-E9. Here, we report the crystal structures of these two VHHs bound to the LCH_N_ domain of BoNT/E1. The structures reveal that these VHHs recognize two distinct epitopes that are partially overlapping with the putative transmembrane regions on H_N_, and therefore could physically block membrane association of BoNT/E1. This is confirmed by our in vitro studies, which show that these VHHs inhibit the structural change of BoNT/E1 at acidic pH and interfere with BoNT/E1 association with lipid vesicles. Therefore, these two VHHs neutralize BoNT/E1 by preventing the transmembrane delivery of LC. Furthermore, structure-based sequence analyses show that the 3-dimensional epitopes of these two VHHs are largely conserved across many BoNT/E subtypes, suggesting a broad-spectrum protection against the BoNT/E family. In summary, this work improves our understanding of the membrane translocation mechanism of BoNT/E and paves the way for developing VHHs as diagnostics or therapeutics for the treatment of BoNT/E intoxication.

## 1. Introduction

Botulinum neurotoxin (BoNT) is one of the most toxic substances known in nature and classified as a Tier 1 select agent by the Centers for Disease Control and Prevention (CDC) in the United States [1,2,3]. There are at least seven immunologically distinct serotypes of BoNT (A–G) that include more than 40 subtypes [4,5,6,7,8]. BoNT/E is one of the four BoNT serotypes (with BoNT/A, BoNT/B, and rarely BoNT/F) known to cause human botulism. There are 12 BoNT/E subtypes (E1–12) identified to date that are produced by *Clostridium botulinum* and *Clostridium butyricum* [9]. BoNT/E intoxication is characterized by a rapid rate of onset and a shorter duration of effect when compared to other BoNTs [10,11,12]. While the majority of BoNT/E intoxication cases are foodborne illnesses caused by consumption of contaminated fish or marine mammal products [13], wound and infant botulism have been reported [14,15]. Recently, BoNT/E is being developed in Phase II clinical trial for therapeutic and aesthetic applications, which increases the potential risk of human exposures to this toxin [16].

The current treatment for adult botulism is an equine heptavalent antitoxin (BAT) in limited supply, which has a short serum half-life and can cause adverse effects including serum sickness and asystole [17,18]. Hence, alternative measures for the treatment of botulism are needed. A cocktail of three monoclonal antibodies (mAbs) against multiple BoNT/E subtypes is currently being developed under Phase I clinical trial [19]. In this study, we aim to develop novel alpaca heavy-chain only antibody (VHH)-based neutralizing agents (VNAs) against BoNT/E as next generation antitoxin therapeutics. We have recently developed a structure-based rational design platform to develop VNAs that show high binding affinity and neutralizing potency against BoNT/A and B [20]. Compared to the mAbs, VHHs are advantageous in their small size, high stability, ease of production, and unique capability to target “hidden” cavities or clefts on the surface of proteins [21,22,23,24].

**Figure 1:**
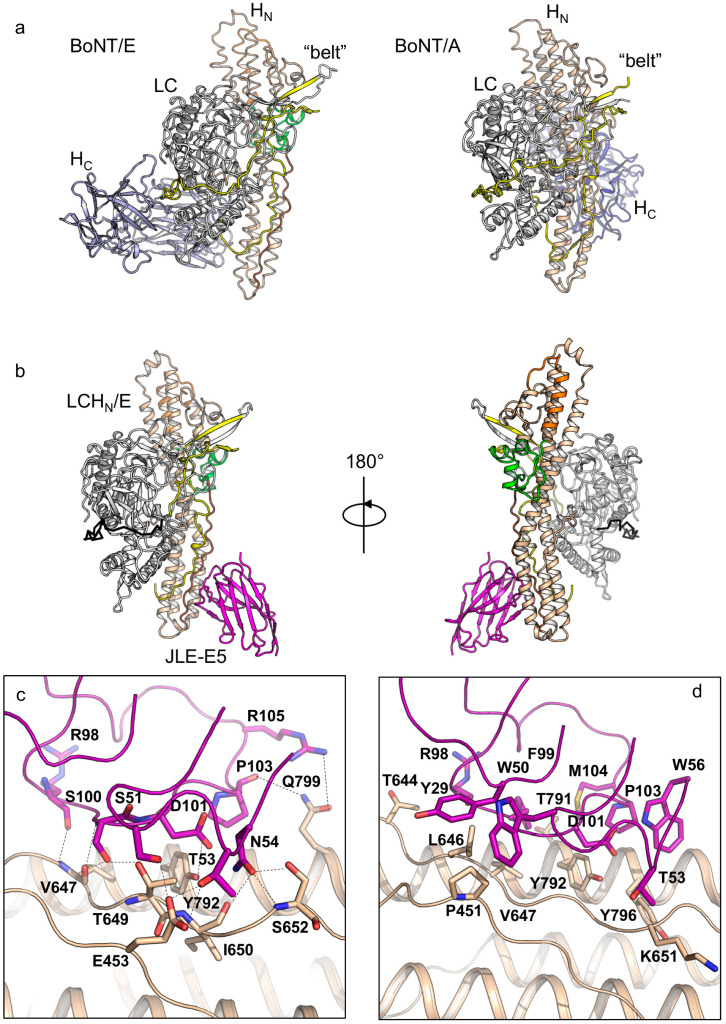
Crystal structure of the LCH_N_/E1–JLE-E5 complex. (**a**) Structures of BoNT/E1 (PDB code 3FFZ) and BoNT/A1 (PDB code 3BTA) holotoxins. (**b**) Cartoon representation of the LCH_N_/E1–JLE-E5 complex. Two orientations of the complex are presented at a rotation of 180° along the y-axis. Coloring scheme: LC, white; “belt”, yellow; H_N_, wheat; channel-forming helix, orange; BoNT-switch, green; amphipathic peptide, brown; JLE-E5, magenta. (**c**,**d**) Molecular interactions between LCH_N_/E1 and JLE-E5. Residues mediating electrostatic interactions (**c**) or hydrophobic interactions (**d**) between LCH_N_/E1 and JLE-E5 are shown as sticks. The complementary-determining regions (CDRs) are drawn in ribbon.

All BoNTs share a tripartite architecture that is composed of a protease domain (LC), a translocation domain (H_N_), and a receptor-binding domain (H_C_) [25]. The toxin is expressed as a single polypeptide chain that is proteolytically cleaved between the LC and H_N_ to form a dichain linked by a disulfide bridge. The three domains are structurally separated in BoNT/A and B [26,27]. However, BoNT/E adopts a unique compact and ‘closed-wing’-like conformation, which may enable more facile interactions among the three domains and therefore lead to its fast onset of intoxication (Figure 1a) [28,29]. The BoNT intoxication mechanism involves three major steps [1,25]. H_C_ recognizes dual receptors, a polysialo-ganglioside (PSG) and a transmembrane glycoprotein (either synaptotagmin or synaptic vesicle protein 2, no protein receptor has been identified for BoNT/C), on presynaptic neurons and triggers toxin internalization [30,31,32,33,34,35]. H_N_ then acts as a protein conduit and delivers the LC across the vesicle membrane to the neuronal cytosol [36,37]. In the cytosol, LC cleaves synaptobrevins, SNAP-25, or syntaxin (all three proteins form the soluble N-ethylmaleimide-sensitive factor attachment protein receptor (SNARE) complex), and their cleavage blocks acetylcholine release at the neuromuscular junction leading to flaccid paralysis [38,39]. It has been shown that antibodies are able to neutralize BoNT action by inhibiting toxin functions at each of these steps [20,40,41,42].

Upon vesicle acidification, the H_N_ of BoNT/E transforms into a LC-conducting channel that shows properties similar to BoNT/A [36,43,44], suggesting the translocation mechanism is conserved across BoNT serotypes. Although the mechanism of channel formation remains poorly characterized, sequence hydropathy analysis of BoNT/E identified three consecutive segments of H_N_ that are predicted to be important for membrane association [45,46]. These segments include a viral-fusion-peptide-like “BoNT-switch” (Q589–E635) [47], the diphtheria toxin-like channel-forming helices (V563–N588) [48], and an extended amphipathic peptide (F636–L654) (Figure 1a) [49]. Prior studies have shown that some neutralizing mAbs and VHHs target the channel-forming helices and interfere with membrane insertion of BoNT/E or A [20,50], but it is not known whether antibodies can neutralize toxin by binding at other putative transmembrane regions.

Here, we report the structural and functional characterization of two neutralizing VHHs raised against BoNT/E1. We determined the crystal structures of these two VHHs in complex with toxin fragments and demonstrated that their mechanisms of neutralization are to inhibit toxin conformational changes at acidic endosomal pH, thus preventing BoNT/E1 membrane interaction. We further show that the binding epitopes of these VHHs are conserved within known BoNT/E subtypes, suggesting that these VHHs merit further evaluation as therapeutic agents for the treatment of BoNT/E intoxication.

## 2. Results and Discussion

### 2.1. The Crystal Structures of the LCH_N_/E1–JLE-E5 and LCH_N_/E1–JLE-E9 Complexes

We recently obtained a large panel of unique BoNT/E1-binding VHHs and identified three VHHs, termed JLE-E5, JLE-E9, and JLE-G6, that neutralize BoNT/E1 intoxication through binding at unique epitopes [51]. Among them, JLE-E5 and JLE-E9 interact with the LCH_N_ domain (residues M1–K845) (Figure S1), a fusion protein composed of the LC and the H_N_ domains of BoNT/E1. We found that JLE-E5 and JLE-E9, which were selected from alpaca immunized with the catalytically inactive BoNT/E1 (ciBoNT/E1), neutralized wild type BoNT/E1 with comparable potency in a mouse neuronal cell-based assay and both bound ciBoNT/E1 with apparent affinities of ≈1 nM EC_50_ in dilution ELISA assays [51]. Recent studies with BoNT/A1 showed that neutralizing VHHs (e.g., ciA-B5 and ciA-H7) targeting LCH_N_/A could counteract toxin action by blocking membrane interaction and delivery of LC [20]. We thus suspected that these VHHs might neutralize BoNT/E1 by a similar mechanism. Furthermore, since the H_N_ is highly conserved among 12 BoNT/E subtypes (97.6% identity), these VHHs could potentially provide broad-spectrum protection against the diverse BoNT/E family [52].

To gain insight into the neutralization mechanisms of JLE-E5 and JLE-E9, we copurified these two VHHs with either ciBoNT/E1 or LCH_N_/E1 in different combinations and carried out crystallization screens. The protein crystals of the ciBoNT/E1–JLE-E5–JLE-E9 and the LCH_N_/E1–JLE-E5–JLE-E9 complexes poorly diffracted X-ray and were not pursued further. After extensive crystallization screening and optimization, we successfully obtained high-quality crystals and determined the structures of the LCH_N_/E1–JLE-E5 and the LCH_N_/E1–JLE-E9 complexes at 2.5 and 3.6 Å resolutions, respectively (Table S1).

**Figure 2:**
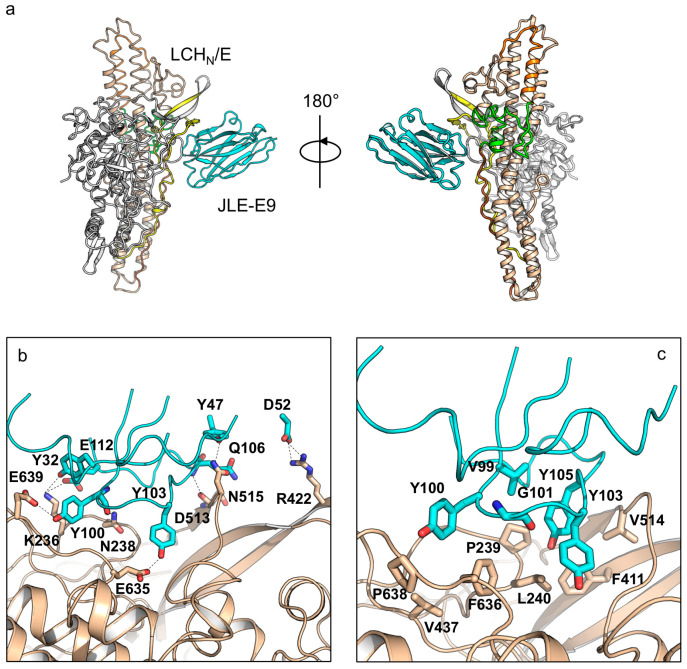
Crystal structure of LCH_N_/E1–JLE-E9. (**a**) Cartoon representation of the LCH_N_/E1–JLE-E9 complex. LCH_N_/E1 is colored as in Figure 1. JLE-E9 is colored in cyan. (**b**,**c**) Molecular interactions between LCH_N_/E1 and JLE-E9. Residues mediating electrostatic interactions (**b**) or hydrophobic interactions (**c**) between LCH_N_/E1 and JLE-E9 are shown as sticks. The CDRs are drawn in ribbon.

Both of these VHHs bind LCH_N_ in a 1:1 ratio, and there are one and four protein complex molecules in one asymmetric unit (ASU) in the crystals of the LCH_N_/E1–JLE-E5 and the LCH_N_/E1–JLE-E9 complexes, respectively (Figure 1b and Figure 2a). Each VHH shows a typical immunoglobulin fold that comprises four framework regions (FR) and three complementary-determining regions (CDR). The absence of H_C_ or the VHH binding does not induce pronounced structural changes in LCH_N_/E1, as the structures of the VHH-bound LCH_N_/E1 domain are highly similar to that of the apo holotoxin (root-mean-square-deviation r.m.s.d = 1.25 Å over 731 Cα atoms, PDB code 3FFZ) [28].

Analysis of the electron density map of LCH_N_/E1 in both structures reveals that the C-terminal loop (residues D832–K845) and the N-terminal “belt” of H_N_ (residues V458–K500) are missing (Figure 1b and Figure S2), indicative of local structural flexibility. However, the inability to detect the 43 amino acids of the belt is unusual because the belt acts as a pseudo-substrate that wraps around LC in all known BoNT structures including BoNT/E1 holotoxin [26,27,53]. Comparing our structures with the holotoxin suggests that the “missing” belt on LCH_N_/E1 could be due to the deletion of H_C_, which interacts with the belt in the context of BoNT/E1 holotoxin [28] and stabilizes the belt and protects LC. This is unique to BoNT/E that adopts a compact closed-wing-like conformation, while all other BoNTs with known structures show an open-wing-like conformation and their H_C_ does not interact with the belt (Figure 1a). Since H_N_ is suggested to be responsible for the rapid onset of BoNT/E intoxication [10], we hypothesize that the exceptional flexibility of the belt may contribute to the speedy translocation of BoNT/E1 by lowering the energy requirement for protein unfolding and LC delivery across the H_N_ channel. Therefore, testing the physiological role of the belt in facilitating BoNT/E translocation is of high interest in future studies.

Further analysis of the crystal packing shows that the LCH_N_/E1 forms a symmetrical homodimer via LC–LC interactions (Figure S2). Prior study also revealed that LC/E forms homodimers in solution as well as in crystal lattice, and the binding mode of LC/E homodimer is nearly identical to that of LCH_N_/E1 homodimer [54]. The holotoxin is not able to form such a homodimer because the H_C_ domain clashes with the interacting partner and sterically blocks the interaction. We observed such LC/E–LC/E interactions in crystals that grew under three different crystallization conditions with two different BoNT/E1 constructs. These findings suggest that LC/E dimerization may represent a natural occurrence rather than a crystallization artifact, but its functional role remains to be clarified.

VHH JLE-E5 binds to the C-terminal boundary of the rod-like H_N_ domain and buries a molecular surface of ≈733.5 Å^2^ per molecule (calculated by PDBePISA v1.51) (Figure 1b) [55]. JLE-E5 binds H_N_E primarily through CDR2 and CDR3 and directly interacts with the belt, the amphipathic segment, and the C-terminal helix of H_N_E (Figure 1c,d, Tables S2 and S3). In CDR2 of JLE-E5, residues S51 and T53 electrostatically interact with E453, while W50 forms an aromatic-proline interaction with P451 of the belt. Residues N54 of CDR2 and R98/S100 of CDR3 form multiple hydrogen bonds with V647, T649, I650, and S652 in the amphipathic segment of H_N_E. In addition, D101, P103, and R105 of CDR3 interact with Y792 and Q799 of the C-terminal helix to stabilize complex formation. Prior studies showed that, upon acidification, the C-terminus of H_N_ interacts with membrane when the toxin engages the PSG receptor (Figure 1a) [28,56]. As a result, the conserved amphipathic peptide transforms from an extended conformation into a transmembrane helix with ion-conducive properties upon membrane insertion [49]. These structural findings suggest JLE-E5 might inhibit the channel formation of BoNT/E1 because it stabilizes H_N_E at its neutral conformation and thus prevents the conformational change of the amphipathic peptide and blocks channel formation.

Structure analysis of JLE-E9 binding to LCH_N_/E1 shows that it uniquely recognizes a composite epitope involving both the LC and the H_N_ domains, which buries an interface area of ≈860 Å^2^ (Figure 2a). A detailed structural analysis revealed that the CDR1, FR2, and CDR3 of JLE-E9 interact with LCH_N_/E1 involving the 230-loop of the LC, the belt, and the molecular switch of H_N_ (Figure 2b,c). Specifically, residues Y32 of CDR1, Y100, and E112 of CDR3 electrostatically interact with K236 and N238 of LC. D52 of FR2 forms two salt bridges with R422 of the belt, while Y47 of FR2 and Q106 of CDR3 form hydrogen bonds with D513 and N515 of the belt. Another interesting finding is that residues Y100 and Y103 of CDR3 interact with E635 of the BoNT-switch and E639 of the amphipathic peptide, two conserved carboxylates in H_N_ that are important for pH sensing (Tables S4 and S5) [37,57]. The LCH_N_/E1–JLE-E9 binding is further strengthened by hydrophobic interactions between residues V99, Y100, G101, Y103, and Y105 of CDR3 with a hydrophobic ridge on BoNT/E1 that is composed of residues P239, L240, F411, V437, V514, F636, and P638. It is believed that the LC and the belt are partially unfolded prior to their entry into the cytosol by translocation through the H_N_ channel (≈15 Å diameter) [36,58]. Therefore, the binding of JLE-E9 might interfere with LCH_N_ unfolding in endosomes and prevent LC translocation. Furthermore, JLE-E9 directly interacts with the pH sensing residues in the BoNT-switch and the amphipathic peptide. These interactions might desensitize the pH-sensing of H_N_ and inhibit its interaction with membrane since the hydrophobic BoNT-switch of H_N_ is believed to be released upon acidification to engage endosomal membrane [47].

### 2.2. JLE-E5 and JLE-E9 Inhibit the Conformational Change of ciBoNT/E1 at Acidic pH

**Figure 3:**
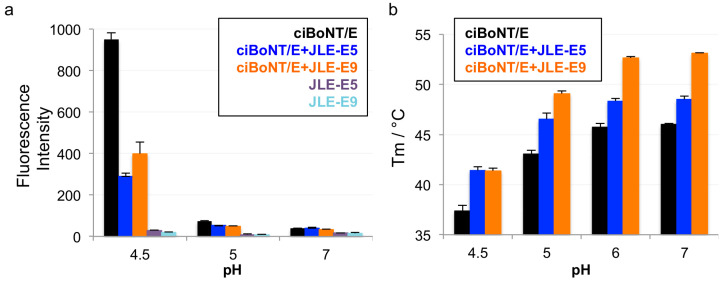
JLE-E5 and JLE-E9 inhibit the conformational change of catalytically inactive BoNT/E1 (ciBoNT/E) at acidic pH. (**a**) ANS fluorescence experiment. ciBoNT/E1 at 0.35 µM was incubated with an equimolar ratio of the indicated VHH in a buffer containing either 50 mM sodium acetate (pH 4.5–5) or HEPES (pH 7.0). All buffers contained 100 mM NaCl and 70 µM ANS. The mean values of fluorescence intensity at 470 nm are shown. (**b**) Thermal stability of ciBoNT/E1, ciBoNT/E1–JLE-E5, and ciBoNT/E1–JLE-E9. The thermal stability of the protein was measured using a fluorescence-based thermal shift assay on a StepOne real-time PCR system (ThermoFisher). Protein melting was monitored using SYPRO Orange dye as the temperature was increased in a linear ramp from 20 to 90 °C. The midpoint of the protein-melting curve (T_M_) was determined using software provided by the instrument manufacturer.

To test our hypothesis that both JLE-E5 and JLE-E9 inhibit the partial unfolding of LC and/or H_N_ at endosomal pH, we examined the unfolding of ciBoNT/E1 using a hydrophobic fluorescence dye ANS [20,47,59]. As shown in Figure 3a, the fluorescence of ANS substantially increased when incubated with ciBoNT/E1 at pH 4.5, but not at pH 7.0 or pH 5.0, indicating ciBoNT/E1 became partially unfolded at pH < 5.0 and agreeing with similar previous studies [59]. The presence of either JLE-E5 or JLE-E9 caused a significant reduction in the ANS fluorescence intensity by ≈60% and ≈72 %, respectively, demonstrating that these VHHs inhibited the structural change of ciBoNT/E1 at acidic pH. We employed an alternative method to measure the effect of these two VHHs on the thermostability of ciBoNT/E1 using a fluorescence-based thermo shift assay (Figure 3b). The melting temperature (T_M_) of ciBoNT/E1 dropped dramatically from 53.1 °C at pH 7 to 37.4 °C at pH 4.5, which indicates ciBoNT/E1 is partially unfolded at low pH (Figure 3b). JLE-E5 and JLE-E9 increased the T_M_ of ciBoNT/E1 at pH 5–7 by 2.5–3.5 °C and 6.0–7.1 °C, respectively. At pH 4.5, both VHHs increased the T_M_ of the holotoxin by ≈4.0 °C. These data consistently demonstrated that these two BoNT/E-neutralizing VHHs stabilize the native conformation of ciBoNT/E1 and thus inhibit the pH-dependent LCH_N_/E1 unfolding, which is an obligatory step for transmembrane delivery of LC/E to the cytosol.

### 2.3. JLE-E5 and JLE-E9 Inhibit the Membrane Interaction of ciBoNT/E

**Figure 4:**
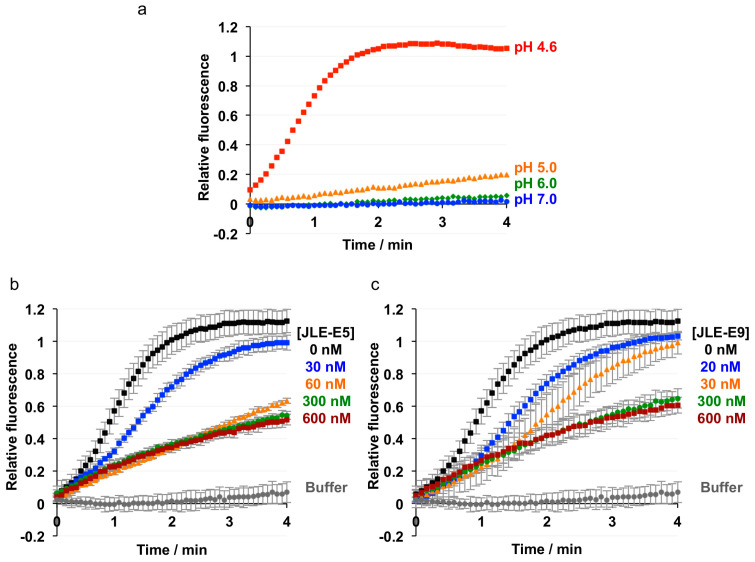
Membrane depolarization assay. Liposomes composed of 70/20/10 mol% of DOPC/DOPS/GT1b were polarized at a negative internal voltage by adding valinomycin in the presence of a transmembrane KCl gradient. Membrane potential was monitored using the fluorescence dye DiOC_2_(3). (**a**) ciBoNT/E1 was added at the indicated buffer pH. The experiment was performed in duplicate. (**b**,**c**) Effect of JLE-E5 (**b**) or JLE-E9 (**c**) on membrane depolarization of ciBoNT/E1. Indicated concentrations of the VHHs were preincubated with ciBoNT/E before the measurement. The data are presented as ± S.D; *n* = 3.

We next investigated the effect of these two neutralizing VHHs on membrane interaction of BoNT/E using a membrane depolarization assay. Similar experiments were successfully employed to study the binding of H_N_A to membrane in recent reports [20,47]. The anionic liposome was prepared containing ganglioside GT1b, the coreceptor for the membrane association of BoNT/E [12]. We found that ciBoNT/E1 triggered an immediate dissipation of valinomycin-induced membrane potential at pH < 5, suggesting that ciBoNT/E1 associated with the artificial lipid bilayer at acidic pH in a way similar to BoNT/A1 [47] (Figure 4a). Notably, the presence of either JLE-E5 or JLE-E9 potently inhibited the ability of ciBoNT/E to depolarize membrane in a VHH concentration-dependent manner (Figure 4b,c). These data collectively suggest that both of these VHHs inhibit the conformational change of BoNT/E1 at endosomal pH and thereby block its channel formation. Recent studies with BoNT/A1 found that a VHH (ciA-B5) that binds in the N-terminal region of H_N_A neutralizes the toxin by inhibiting membrane insertion of H_N_A and blocking channel formation. However, unlike JLE-E5 and JLE-E9, ciA-B5 does not affect the low-pH triggered structural rearrangement of BoNT/A. These antibodies thus likely trap the toxins in different intermediate states through the course of channel formation of H_N_. VHHs have been previously applied to study the dynamic conformations of membrane proteins [60]. Our work thus provides valuable tools for future mechanistic studies of channel formation mechanisms for BoNTs.

### 2.4. The JLE-E5- and JLE-E9-Binding Epitopes are Conserved Across Several BoNT/E Subtypes

**Figure 5:**
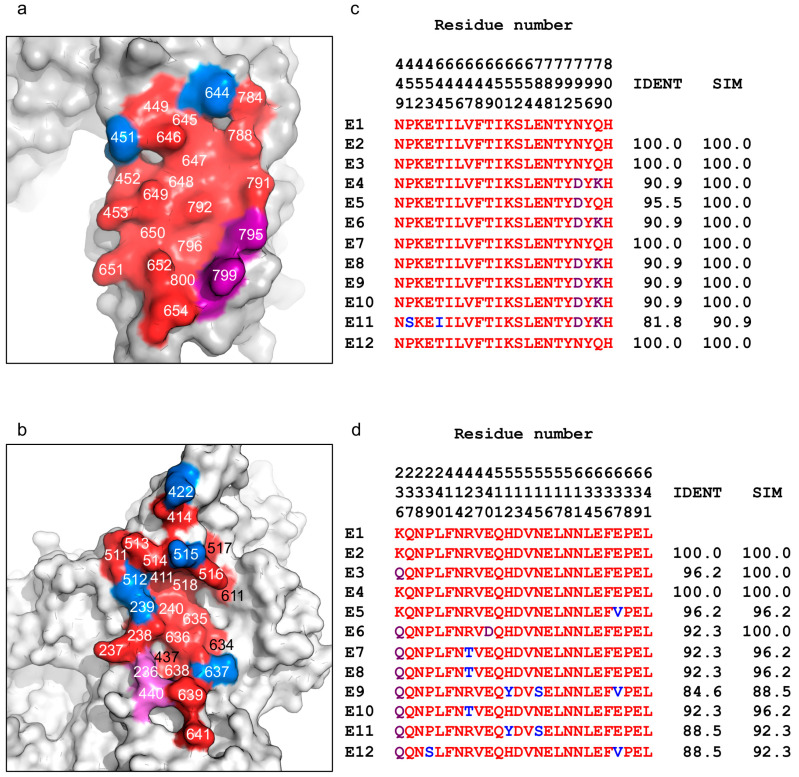
Sequence conservation of the VHH-binding epitopes across BoNT/E subtypes. (**a**,**b**) Sequence conservation of (**a**) JLE-E5- and (**b**) JLE-E9-binding epitopes are plotted on the LCH_N_/E1 structure. Identical, conserved, and variable residues at the binding interface are colored red, purple, and blue, respectively. (**c**,**d**) Amino acid sequence alignment among all BoNT/E subtypes. Only amino acids forming the JLE-E5 (**c**) or JLE-E9-binding (**d**) epitopes on BoNT/E are presented with residue numbers as indicated (displayed vertically, top down, for each residue position). The percentage of sequence identity (IDENT) and similarity (SIM) are listed. Sequence alignments were performed using Clustal Omega.

Twelve naturally occurring BoNT/E subtype variants have been reported to date, which pose significant challenges to developing broad-spectrum antitoxins against this toxin serotype. Fortunately, as shown in Figure 5, sequence analyses show that both JLE-E5 and JLE-E9-binding epitopes are quite highly conserved across the different BoNT/E subtypes, with sequence similarities of >90% and >88%, respectively. Notably, the JLE-E5-binding epitopes is identical in four other subtypes (E2, E3, E7, and E12), while a conserved mutation of N795D in subtype E5 and an additional charge replacement of Q799K in E4, E6, and E8–E10 may mildly affect JLE-E5 interactions (Figure 5a,c). The only exception is BoNT/E11, which has two unique amino acids at S451 and I644, which may weaken JLE-E5 binding. Hence, JLE-E5 is likely to bind well to most or all of the BoNT/E subtypes. The JLE-E9 binding epitope is less conserved compared to JLE-E5. This epitope is identical within subtypes E2 and E4 while there are 1–4 amino acid substitutions in the other BoNT/E subtypes that may weaken their recognition and neutralization by JLE-E9 (Figure 5b,d). Notably, our structures show that the epitopes recognized by these two VHHs are closely apposed on BoNT/E, thus making it feasible to create a simultaneous binding bivalent JLE-E5/JLE-E9 VNA by connecting them via a short peptide linker. Recent studies showed that bivalent VNAs with component VHHs that can bind simultaneously to the same toxin molecule have superior affinities and potency to neutralize toxin [20]. As shown in Tremblay et al. [51], a designer VNA optimized for simultaneous binding of JLE-E5 and JLE-E9 to BoNT/E1 displayed significantly improved antitoxin potency compared to a VNA that has the same VHH components but was not optimized for simultaneous binding. Beyond its superior antitoxin potency, this VNA will likely have strong binding to all BoNT/E subtypes due to the synergy between the two VHHs that should counteract a few subtle amino acid variations at individual epitopes, and therefore promises to be a broad-spectrum antitoxin against most or all BoNT/E subtypes.

## 3. Conclusions

In summary, we report the crystal structures of two BoNT/E-neutralizing VHHs, JLE-E5 and JLE-E9, in complex with LCH_N_/E1 at 2.5 and 3.6 Å resolutions, respectively. Our structural data show that these VHHs recognize novel epitopes at the interface of the LC–H_N_ domains via direct interactions with the membrane-interacting segment of the H_N_. Therefore, they are able to inhibit the acidic pH-triggered conformational change of BoNT/E1 and preclude its interaction with membrane. This hypothesis is supported by our biochemical studies showing that both VHHs stabilize BoNT/E1 at low pH and inhibit the binding of the toxin to lipid vesicles. Sequence analysis of the JLE-E5- and JLE-E9-binding epitopes reveal that these epitopes are quite conserved among BoNT/E subtypes, suggesting these VHHs are valuable building blocks for developing multivalent VNAs with broad protection capability. This study furthers our understanding of the membrane translocation of BoNTs and provides novel insights into antibody neutralization mechanisms targeting BoNT/E and potentially other BoNT subtypes.

## 4. Materials and Methods

### 4.1. Cloning, Expression, and Purification of Recombinant Proteins

Catalytically inactive BoNT/E1 (ciBoNT/E1) that carries three mutations (H212A/E213A/H216A), LCH_N_/E1 (M1–K845), VHH JLE-E5, and JLE-E9 were cloned into pGEX-6p-1 vector following the N-terminal GST and a PreScission cleavage site. The proteins were expressed in *Escherichia coli* strain BL21-Star (DE3) (Invitrogen). Transformed bacteria were grown at 37 °C in LB medium in the presence of ampicillin. Expression was induced with 1 mM isopropyl-b-D-thiogalactopyranoside (IPTG) when OD600 reached ≈0.6–0.8. Temperature was then decreased to 18 °C and expression was continued for 16 h. Cells were harvested by centrifugation and stored at −20 °C until use.

For protein purification, bacteria were resuspended in a buffer containing 50 mM Tris (pH 8.0), 400 mM NaCl, and 0.4 mM PMSF and lysed by sonication. The GST-tagged proteins were purified using glutathione Sepharose 4B resins (GE Healthcare, Marlborough, MA, USA) and eluted from the resins after on-column cleavage in PBS using PreScission protease. The proteins were further purified by Superdex-200 Increase or Superdex-75 size exclusion chromatography (SEC; GE Healthcare) in 150 mM NaCl and 10 mM HEPES (pH 7.4). The LCH_N_/E1–JLE-E5 and LCH_N_/E1–JLE-E9 complexes were prepared by mixing the purified LCH_N_/E1 and VHH at a molar ratio of 1:1.5 for 1 h on ice, followed by purification using Superdex-200 Increase SEC (10 mM HEPES, pH 7.4, and 150 mM NaCl). Each protein was concentrated to ≈5 mg/mL using Amicon Ultra centrifugal filters (Millipore, Burlington, VT, USA) and stored at −80 °C until further characterization or crystallization.

### 4.2. Membrane Depolarization Assay

Large unilamellar vesicles (LUV) were prepared as previously described [47]. Briefly, lipids (1,2-dioleoyl-sn-glycero-3-phospho-L-serine (DOPS) and 1,2-dioleoyl-sn-glycero-3-phosphocholine (DOPC)) (Avanti Polar Lipid, Alabaster, USA) were dissolved in chloroform while GT1b trisodium salt (Santa Cruz Biotechnology, Dallas, TX, USA) was dissolved in methanol. The lipids at the indicated molar ratios were mixed and then dried under nitrogen gas and placed under vacuum overnight. The dried lipids were rehydrated and subjected to 5–10 rounds of freezing and thawing cycles. Liposomes were prepared by extrusion through a 200 nm pore membrane using an Avanti Mini Extruder according to the manufacturer’s instructions.

Liposomes composed of 70/20/10 mol% of DOPC/DOPS/GT1b were prepared in 100 mM KCl, 1 mM NaCl, and 10 mM HEPES (pH 7.0). To create a trans-negative membrane potential (−117 mV), liposomes were diluted in 100 mM NaCl, 1 mM KCl, and 10 mM sodium acetate (pH 4.6). Membrane potential was monitored using 1 µM 3,3′-diethyloxacarbocyanine iodide (DiOC2(3)) (Sigma-Aldrich, St. Louis, MO, USA) [61]. Valinomycin was added at time 0-s to give a final concentration of 5 µM. At 60-s, 100 nM of ciBoNTE, preincubated with 0–600 nM of JLE-E5 or JLE-E9, was added and the fluorescence intensity at 535 nm was monitored for 4 min with excitation at 488 nm. The reaction was stopped by adding 2 µM of gramicidin from *Bacillus anerinolyticus* (Sigma-Aldrich, St. Louis, USA). The fluorescence change relative to the maximal change in the presence of gramicidin was calculated as (F − F_initial_)/(F_final_ − F_initial_). The experiments were repeated three times independently.

### 4.3. 8-Anilinonaphthalene-1-Sulfonic Acid Binding Assay

ciBoNT/E, ciBoNT/E–VHH, or VHH was incubated at ≈0.35 µM with 70 µM ANS for 30 min at 37 °C in either 50 mM sodium acetate (pH 4.5–5.0) or 50 mM HEPES (pH 7). All buffers contained 100 mM NaCl. Fluorescence intensity was recorded at 25 °C using a SpectraMax M2e spectrophotometer (Molecular Devices, San Jose, CA, USA) with excitation at 370 nm and emission at 470 nm. The fluorescence intensity was corrected by subtraction of background fluorescence from ANS in a buffer without protein. Error bars indicate SD of three replicate measurements.

### 4.4. Thermal Denaturation Assay

The thermal stability of ciBoNT/E or ciBoNT/E–VHH was measured using a fluorescence-based thermal shift assay on a StepOne real-time PCR machine (Life Technologies, Carlsbad, CA, USA). ciBoNT/E with or without VHH was incubated for 30 min in a buffer containing 0.1 M NaCl combined with 50 mM sodium acetate (pH 4.5–5.0) or 50 mM sodium citrate (pH 6) or 50 mM HEPES (pH 7.0). Immediately before the experiment, the protein (0.7 µM) was mixed with the fluorescent dye SYPRO Orange (Sigma-Aldrich). The samples were heated from 20 to 90 °C in a standard ramp rate of 1.5 °C/min. The midpoint of the protein-melting curve, the T_M_, was determined using the analysis software provided by the instrument manufacturer. The experiment was performed in triplicate.

### 4.5. Crystallization

Initial crystallization screens were performed using a Gryphon crystallization robot (Art Robbins Instruments, Mountain View, CA, USA) and high-throughput crystallization screen kits (Hampton Research and Qiagen). Extensive manual optimizations were performed at 18 °C. The best single crystals of LCH_N_/E1–JLE-E5 were grown by the sitting-drop vapor diffusion method at a protein concentration of 5 mg/mL with a reservoir solution containing 0.15 M Ammonium sulfate, 14% PEG 4000, 0.1 M Hepes (pH 7.0) when proteins were mixed with reservoir solution at 1:1 (*v*/*v*) ratio. Crystals of LCH_N_/E1–JLE-E9 were obtained by the sitting-drop vapor diffusion method at a protein concentration of 6 mg/mL with a reservoir solution containing 12% PEG 20K, 0.1 M sodium citrate (pH 5.8) when proteins were mixed with reservoir solution at 2:1 (*v*/*v*) ratio.

### 4.6. Data Collection and Structure Determination

All crystals were cryoprotected in their original mother liquor supplemented with 15–20% (*v*/*v*) ethylene glycol. The X-ray diffraction data were collected at 100 K at the NE-CAT beamline 24-ID-E, Advanced Photon Source (APS). The data were processed with XDS as implemented in RAPD (https://github.com/RAPD/RAPD) [62]. Data collection statistics are summarized in Table S1. Structures of the LCH_N_/E1–JLE-E5 and LCH_N_/E1–JLE-E9 complexes were determined by molecular replacement using the Phaser software [63] with LCH_N_/E (PDB code 3FFZ) [28] and the homology models of JLE-E5 and JLE-E9 that were built based on a VHH in PDB 5L21 [41] as the search models. Manual model building and refinement were performed in COOT [64], PHENIX [65], and CCP4 suite [66] in an iterative manner. The refinement progress was monitored with the free R value using a 5% randomly selected test set [67]. The structures were validated through MolProbity [68] and showed excellent stereochemistry. Structural refinement statistics are listed in Table S1. All structure figures were prepared with PyMol (http://www.pymol.org).

### 4.7. Accession Code

Atomic coordinates and structure factors for the LCH_N_/E1–JLE-E5 and LCH_N_/E1–JLE-E9 complexes have been deposited in the Protein Data Bank under accession codes 7K84 and 7K7Y, respectively.

## Supplementary Materials

The following are available online at https://www.mdpi.com/2072-6651/12/10/616/s1, Figure S1: VHH JLE-E5 and JLE-E9 interact with LCH_N_/E1, Figure S2: LCH_N_/E1 interacts with symmetry molecule via LC/E–LC/E homo-dimerization, Table S1: Data collection and refinement statistics, Table S2: Buried surface area of JLE-E5 in complex with LCH_N_/E1, Table S3: Buried surface area of LCHN/E1 in complex with JLE-E5, Table S4: Buried surface area of JLE-E9 in complex with LCH_N_/E1, Table S5: Buried surface area of LCH_N_/E1 in complex with JLE-E9.
