# Supplementary material for: Two VHH Antibodies Neutralize Botulinum Neurotoxin E1 by Blocking Its Membrane Translocation in Host Cells
Source: Toxins (Basel). 2020 Sep 27;12(10):616. doi: 10.3390/toxins12100616 (PMC7599855; doi:10.3390/toxins12100616)
Supplement: Supplementary file 1 [file toxins-12-00616-s001.pdf]

Supplementary Materials: Two VHH Antibodies Neutralize Botulinum Neurotoxin E1 by Blocking its Membrane Translocation in Host Cells

Kwok-Ho Lam, Kay Perry, Charles B. Shoemaker and Rongsheng Jin

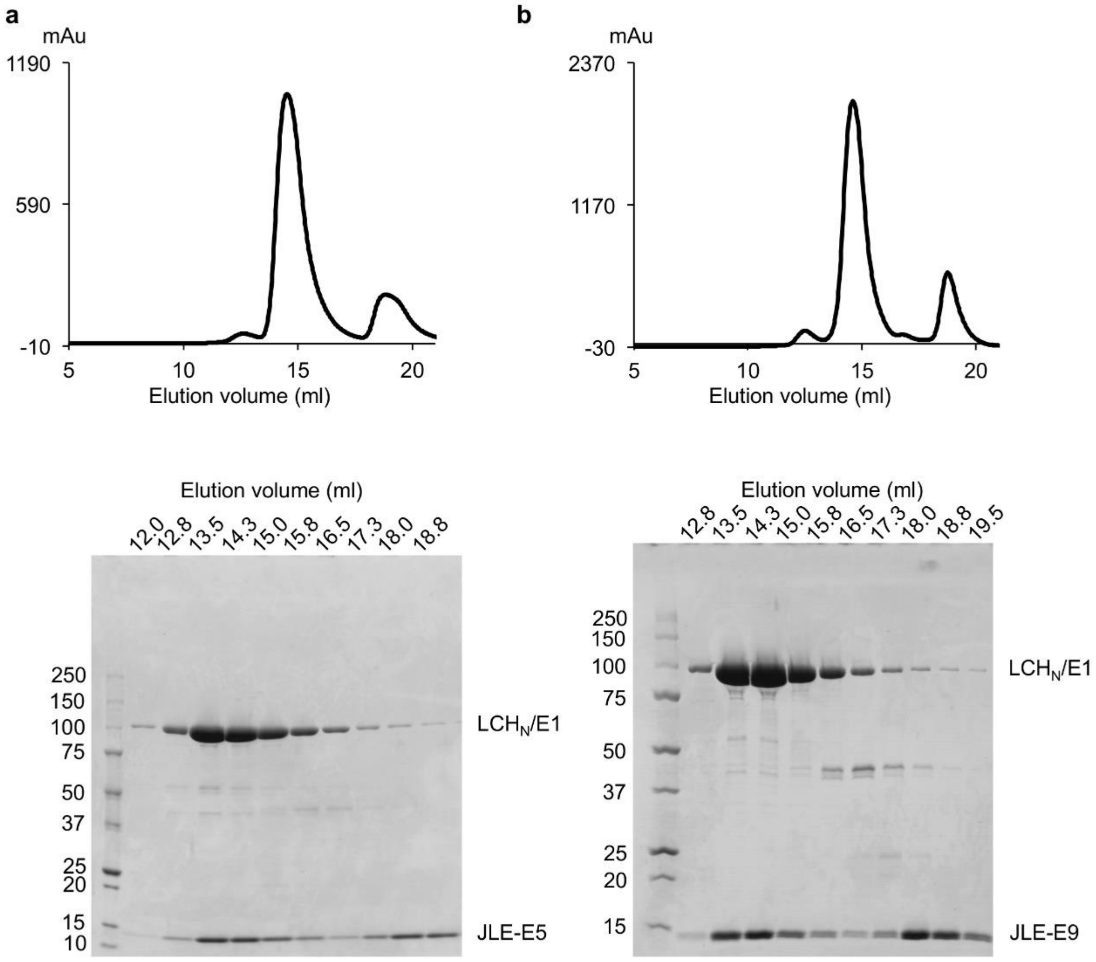

Figure S1. VHH JLE-E5 and JLE-E9 interact with LCHN/E1. Gel filtration analysis. LCHN/E1 was pre-incubated with JLE-E5 (**a**) or JLE-E9 (**b**) at 4 °C for 1 hour followed by separation with S200-SEC. The peak fractions were analyzed by SDS-PAGE.

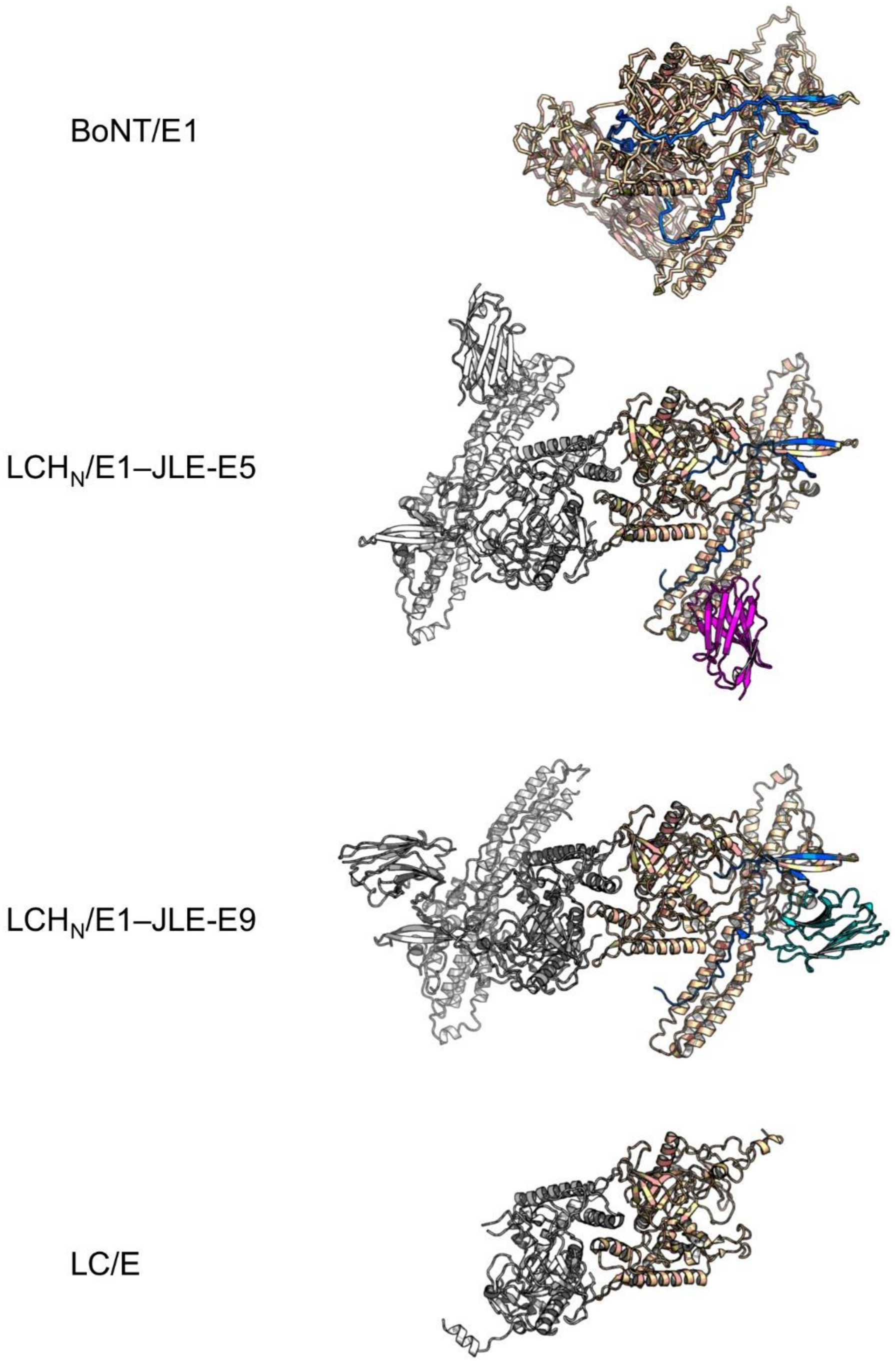

Figure S2. LCHN/E1 interacts with symmetry molecule via LC/E–LC/E homo-dimerization. Cartoon representations of BoNT/E holotoxin (PDB code 3FFZ), LCHN/E1–JLE-E5, LCHN/E1–JLE-E9, and LC/E (PDB code 1T3A). BoNT/E is colored in wheat while the belt is highlighted blue. Note that a portion of the belt is missing in LCHN/E1–VHH structures. The LCHN/E1 or LC/E interacts with symmetry molecule (white) related by a two-fold rotation along the z-axis.

Table S1. Data collection and refinement statistics.

	LCH_N/E1–JLE-E5	LCH_N/E1–JLE-E9
**Data collection**		
Space group	P 1 2 1	P 21 21 21
Cell dimensions		
a, *b*, *c* (Å)	96.09, 49.88, 133.51	109.04, 208.65, 210.51
α, β, γ (°)	90, 101.04, 90	90, 90, 90
Resolution (Å)	131.04–2.50 (2.59–2.50)[a]	148.19 - 3.6 (3.70–3.60)
R_{pim}	0.093 (0.684)	0.11 (0.960)
CC1/2	0.993 (0.661)	0.997 (0.309)
I/σ(I)	6.3 (1.5)	6.6 (0.9)
Completeness (%)	96.3 (95.2)	98.4 (99.5)
Redundancy	2.8 (2.9)	3.8 (3.9)
**Refinement**		
Resolution (Å)	65.52–2.50	74.10–3.60
No. reflections	41860	55219
Reflections used for R_{free}	2091	2699
R_{work}/R_{free}	0.234 / 0.242	0.261 / 0.288
No. atoms	7397	28699
Protein	7305	28699
Ligand/ion	16	-
Water	76	-
B-factors	73.70	123.9
Protein	73.70	123.9
Ligand/ion	99.60	-
Water	66.30	-
R.m.s. deviations		
Bond lengths (Å)	0.007	0.002
Bond angles (°)	1.13	0.52

One crystal was used for each structure. [a] Statistics for the highest-resolution shell are shown in parentheses.

Table S2. Buried surface area of JLE-E5 in complex with LCHN/E1.

JLE-E5 residue	Accessible Surface Area (Å^2)	Buried Surface Area (Å^2)	Bond type
Y29	87.60	50.71	
S49	6.54	6.54	
W50	88.55	32.04	
S51	70.43	18.96	H
T53	111.21	56.27	
N54	57.51	56.48	H
T55	67.50	2.77	H
W56	82.28	70.38	
H97	102.37	45.46	
R98	137.96	85.34	H
F99	75.47	70.77	
S100	69.99	68.33	H
D101	35.62	35.50	H
P103	46.86	46.86	H
M104	76.48	65.61	
R105	124.03	27.66	H

Table S3. Buried surface area of LCHN/E1 in complex with JLE-E5.

LCHN/E1 residue	Accessible Surface Area (Å^2)	Buried Surface Area (Å^2)	Bond type
N449	35.47	16.07	
P451	116.85	63.11	
K452	99.32	2.33	
E453	126.98	49.88	H
T644	80.81	54.85	
I645	4.92	2.94	
L646	97.10	85.72	
V647	31.94	31.78	H
F648	9.17	4.41	
T649	65.46	61.41	H
I650	4.92	4.92	H
K651	110.45	22.46	
S652	68.09	41.76	H
L654	111.63	5.51	
E784	50.87	7.54	
N788	65.51	42.95	
T791	79.56	25.94	
Y792	67.65	67.49	H
N795	80.94	40.49	
Y796	15.64	15.64	
Q799	130.18	76.81	H
H800	3.28	3.28	

H, Hydrogen bond.

Table S4. Buried surface area of JLE-E9 in complex with LCH_N/E1.

JLE-E9 residue	Accessible Surface Area (Å^2)	Buried Surface Area (Å^2)	Bond type
Q1	128.06	20.04	H
R27	114.37	13.01	
T28	46.94	19.73	
S31	47.42	16.25	
Y32	12.75	7.91	
Y47	32.59	5.98	H
A50	1.17	0.33	
N52	15.39	11.31	H
S57	43.28	7.12	
F59	100.30	52.71	
D62	149.21	42.33	HS
V99	20.23	17.56	
Y100	142.10	130.42	H
G101	25.70	12.31	
R102	132.58	42.35	H
Y103	144.27	133.57	H
T104	14.31	12.80	H
Y105	109.24	106.04	
Q106	117.24	60.80	H
S107	51.06	40.51	H
K109	196.70	9.03	
S110	44.03	37.89	
Y111	32.75	2.01	
E112	80.51	46.37	S
Y113	63.05	8.72	

Table S5. Buried surface area of LCH_N/E1 in complex with JLE-E9.

LCH_N/E1 residue	Accessible Surface Area (Å^2)	Buried Surface Area (Å^2)	Bond type
K236	87.38	57.72	S
Q237	163.56	32.10	
N238	56.21	41.08	H
P239	51.95	44.32	
L240	26.29	24.27	
F411	5.47	5.47	
N414	80.66	7.99	
R422	172.43	38.85	HS
V437	4.35	1.67	
E440	113.16	31.95	HS
D508	109.27	5.82	
Q511	95.07	13.38	H
H512	42.09	35.28	
D513	115.37	68.51	H
V514	44.10	40.79	
N515	142.52	119.54	H
E516	130.23	85.43	H
L517	33.71	7.96	
N518	15.50	14.86	
V519	3.16	1.84	
L634	16.93	6.32	

E635	56.64	46.07	H
F636	68.96	52.78	
E637	79.73	6.14	H
P638	33.53	12.39	
E639	147.94	53.24	H

H, Hydrogen bond; S, Salt bridge
